# How the crosstalk between innate immune sensors and metabolic pathways affect the outcome of *Brucella abortus* infection?

**DOI:** 10.3389/fmicb.2022.995219

**Published:** 2022-08-11

**Authors:** Sergio C. Oliveira, Erika S. Guimarães

**Affiliations:** ^1^Departamento de Bioquímica e Imunologia, Instituto de Ciências Biológicas, Universidade Federal de Minas Gerais, Belo Horizonte, Minas Gerais, Brazil; ^2^Departamento de Genética, Ecologia e Evolução, Programa de Pós-Graduação, Instituto de Ciências Biológicas, Universidade Federal de Minas Gerais, Belo Horizonte, Minas Gerais, Brazil

**Keywords:** innate immunity, immunometabolism, *Brucella*, STING, AIM2, MyD88

## Introduction

*Brucella* is an important human and animal bacterial pathogen that can survive in macrophages causing chronic infections (Roop et al., 2009). Innate immunity is the first line of defense against pathogens such as *Brucella*. TLR9, AIM2, MyD88, and STING are important receptors and adaptor molecules that are involved in protective responses against *Brucella* infection (Macedo et al., 2008; Gomes et al., 2016; Costa Franco et al., 2018, 2019). Microbial pathogens such as *Brucella* use different host cell energy sources to replicate intracellularly. Erythritol, glutamic acid, and glucose are efficiently metabolized by *Brucella* (Anderson and Smith, 1965). Macrophages are central population of cells of innate immunity; however, it is clear that macrophage phenotypes are difficult to categorize. They can be oversimplified into two major profiles, a pro-inflammatory (M1) and an anti-inflammatory (M2) subsets (Viola et al., 2019). Previously, it was reported that *Brucella abortus* survives and replicates preferentially in anti-inflammatory (M2), which are more abundant during chronic infection (Xavier et al., 2013). Glucose uptake was involved in *B. abortus* replication in M2 macrophages during chronic infection. Inactivation of *Brucella* glucose transporter gluP lead to reduced bacterial survival in macrophages and mouse susceptibility to infection. Additionally, stimulation of peroxisome proliferator-activated receptor γ (PPARγ) results in enhanced availability of glucose for *Brucella* in M2 macrophages augmenting bacterial replication (Xavier et al., 2013).

Macrophages and dendritic cells can undergo a change in energy metabolism by shutting down oxidative phosphorylation and increasing the rate of aerobic glycolysis in a pathway termed as the Warburg effect (Kelly and O'Neill, 2015). Macrophages from humans infected with *Brucella abortus* undergo a Warburg-effect metabolic change to an aerobic glycolytic profile (Czyz et al., 2017). Czyz et al. (2017) demonstrate that inhibition of host glycolysis and lactate production by using 3-BPA and NHI-2 reduced bacterial replication intracellularly without affecting *Brucella* growth. Metabolic reactions such as glycolysis, the Krebs cycle, fatty acid metabolism, and nitrogen metabolism are critical pathways that host cells undergo to combat several pathogens (Escoll and Buchrieser, 2018). In this opinion article, we connected the interplay of host innate immune recognition of the intracellular bacteria *Brucella* with recent findings in immunemetabolism and how this findings can impact on the outcome of infection.

## TLRs and immunometabolism

**Figure 1:**
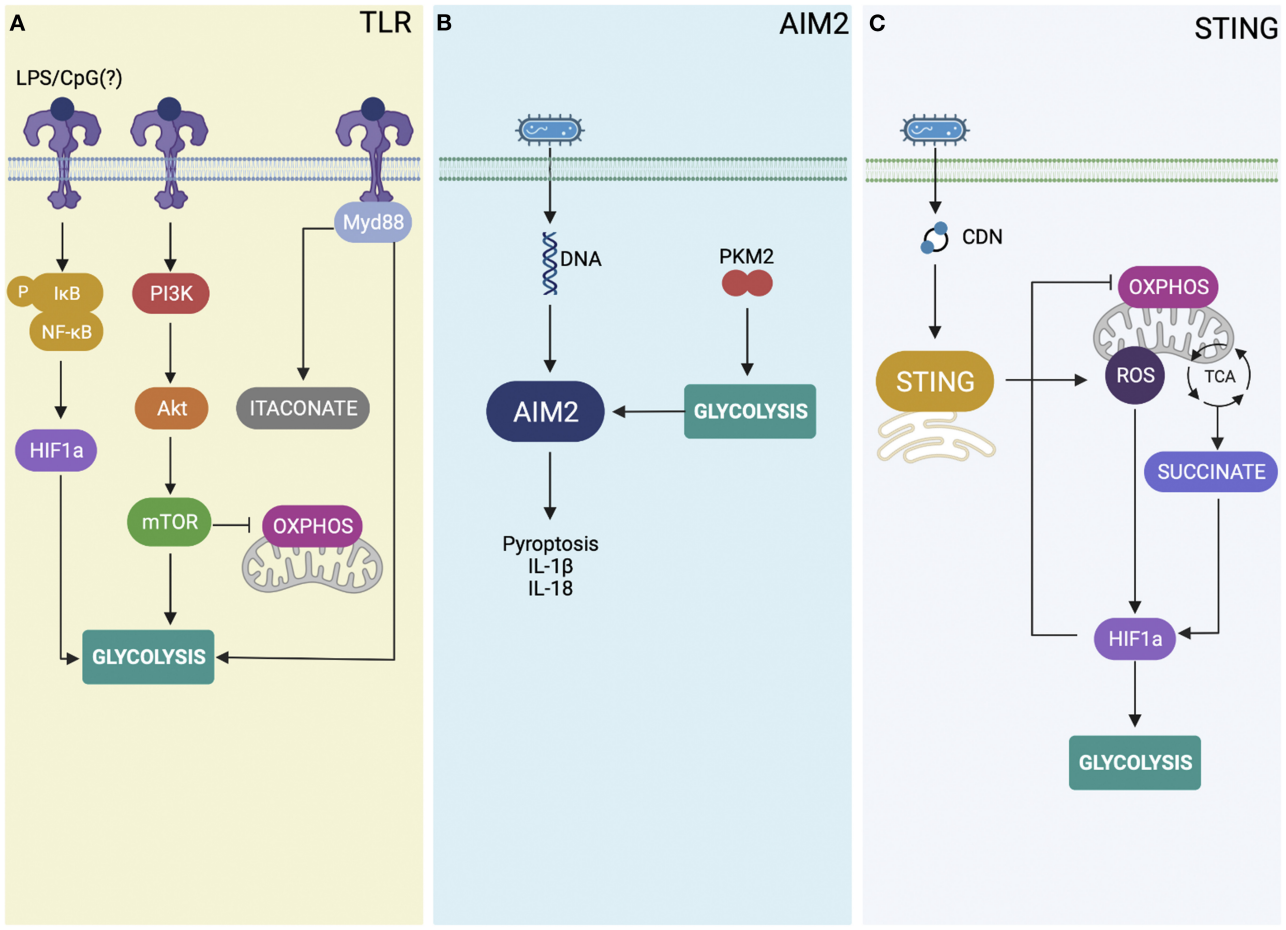
Overview of the role of innate immune receptors in immunometabolism. **(A)** TLR (toll-like receptors) stimulation activates the NF-κB (nuclear factor kappa B) pathway and the transcription factor HIF-1α (hypoxia-inducible factor-1α), thereby inducting the metabolic reprogramming toward glycolysis. Additionally, TLR stimulation also activates the PI3K-AKT-mTOR (phosphatidylinositol 3-kinase-protein kinase B-mechanistic target of rapamycin) pathway, a signaling pathway that plays a critical role in inducing the metabolic reprogramming and glycolysis. Further, *Brucella* activates TLRs signaling through MyD88-dependent glycolysis that results in itaconate production and restriction of *Brucella* infection. **(B)** Bacterial DNA and the M2 isoform of pyruvate kinase muscle 2 (PKM2)-dependent aerobic glycolysis activates AIM2 leading to IL-β secretion and pyroptosis. **(C)** During *B. abortus* infection, STING activation increases intracellular succinate levels and mROS (mitochondrial reactive oxygen species) production that contributes to HIF-1α stabilization. HIF-1α drives the metabolic reprogramming in infected macrophages, increasing glycolysis and reducing OXPHOS (mitochondrial oxidative phosphorylation).

TLR activation by microbial products can provide signal for metabolic shift in immune cells. TLR signaling activates the NF-κB and HIF-1α transcription factors inducing transcriptional reprogramming toward the glycolytic gene expression profile in macrophages (Krawczyk et al., 2010). It is already established that LPS binding to TLR4 activate multiple downstream metabolic pathways in different Gram-negative bacterial infections (Pan et al., 2022). However, in the case of *Brucella* infection, TLR9 is the most single TLR associated with host protection against infection, suggesting the bacterial DNA as an important bacterial agonist (Gomes et al., 2016). *B. abortus* or its DNA induced activation of MAPK/NF-κB pathways and production of IL-12 and TNF-α by macrophages partially dependent on TLR9 (Gomes et al., 2016). Bacterial LPS leads to HIF-1α and PI3K-AKT-mTOR activation leading to glycolysis and an inflammatory macrophage state (Pan et al., 2022; Figure 1A). However, if *Brucella* DNA *via* TLR9 induces this metabolic shift in macrophages yet to be determined. All TLRs except TLR3, signal through the adaptor molecule myeloid differentiation factor 88 (MyD88; Adachi et al., 1998). Activation of the TLR/MyD88 axis in host cells can promote glycolysis and glucose consumption (Lachmandas et al., 2016). Previously, our group has demonstrated that MyD88-dependent signaling is critical to *Brucella* control in mice leading to dendritic cell maturation and IL-12 production (Macedo et al., 2008). Others have shown that enhanced replication of *B. abortus* in M2 macrophages require the function of *Brucella* glucose transporter gluP metabolizing host glucose (Xavier et al., 2013). More recently, Lacey et al. determined whether MyD88-dependent host glycolysis could be involved in control of *B. melitensis* infection (Lacey et al., 2021). Their findings suggest that glucose restriction induced by MyD88 signaling pathway was important for control of *B. melitensis* infection *in vivo*. Additionally, they found that itaconate production is dependent on MyD88 and this metabolite can reduce *Brucella* replication and modulate pro-inflammatory cytokine responses. It would be interest to see whether the activation of a single TLR, such as TLR9 would be able to trigger glycolysis and itaconate production in host cells infected with *Brucella*.

## AIM2 inflammasome and glycolysis

AIM2 was identified as the receptor involved in inflammasome activation in response to the recognition of cytosolic DNA during bacterial infections (Rathinam et al., 2010) leading to the production of IL-1β and IL-18, and pyroptosis (Hornung et al., 2009). Previously, our group has demonstrated that AIM2 senses *Brucella* DNA in dendritic cells to induce pyroptosis (Costa Franco et al., 2019). Dendritic cells from AIM2-deficient animals infected with *B. abortus* showed reduced caspase-1 processing and diminished IL-1β secretion. AIM2-deficient animals also displayed reduced resistance to *B. abortus* infection, and this susceptibility was associated with defective IL-1β secretion and reduced IFN-γ T cell responses. However, the influence of AIM2 activation on host cell immunometabolism during *Brucella* infection is unknown.

The M2 isoform of pyruvate kinase muscle 2 (PKM2)-dependent aerobic glycolysis induces IL-1β secretion in LPS-activated macrophages (Palsson-McDermott et al., 2015). However, still to be determined whether PKM2-induced glycolysis regulates IL-1β secretion by modulating inflammasome activation. Studies by Xie et al. showed that PKM2-induced glycolysis promotes AIM2 inflammasome activation by producing lactose to modulate eukaryotic translation initiation factor 2 alpha kinase 2 (EIF2AK2, also termed PKR) phosphorylation in macrophages during sepsis (Xie et al., 2016). The authors also showed that blocking the PKM2-EIF2AK2 hub using target inhibitors can reduce inflammasome activation and protect mice from sepsis. Furthermore, genetic deletion of PKM2 in myeloid cells reduces inflammasome activation and protects animals against death by septic shock. Besides AIM2 activation induced by PKM2-mediated glycolysis, Cho et al. demonstrated a novel mechanism that links glucose transporter 1 (GLUT1)-mediated glycolysis and AIM2 to modulate lung fibrogenesis caused by *Streptococcus* infection (Cho et al., 2020). Glut1-deficient mice showed reduced morbidity and collagen levels in bleomycin-induced lung fibrosis upon *Streptococcus pneumoniae* infection. Reduced AIM2 inflammasome activation by poly(dA:dT) was also observed in Glut1-KO macrophages. It is possible that glycolysis and enhanced expression of GLUT1 marker observed in *Brucella*-infected macrophages (Gomes et al., 2021) triggers AIM2-inflammasome activation and IL-1β secretion and helps to control bacterial infection as presented in Figure 1B. However, this hypothesis has to be proven by further experiments.

## STING paving the way to inflammatory macrophages

STING is an adaptor molecule that together with cGAS is critical to sense cytosolic DNA from different pathogens (Ishikawa and Barber, 2008). During intracellular bacterial infection such as *Brucella*, activation of STING can be accomplished by two different pathways. STING can directly recognize bacterial cyclic dinucleotides (CDNs; Burdette et al., 2011), or senses DNA *via* cGAS triggering cGAMP synthesis and then activating STING as a secondary receptor (Sun et al., 2013). Previously, we have demonstrated that STING is important to control *Brucella* infection in macrophages and *in vivo* but not the receptor cGAS (Costa Franco et al., 2018). More recently, we reveal the mechanisms by which STING induces an inflammatory macrophage profile following *Brucella* infection (Gomes et al., 2021). This metabolic shift induced by STING helps to stabilize the hypoxia-inducible factor-1 alpha (HIF-1α), a transcription factor involved in cellular metabolism and innate immune functions. HIF-1α stabilization reduced oxidative phosphorylation and increases glycolysis during infection with *B*. *abortus*. This metabolic reprogramming leads to augmented nitric oxide production, inflammasome activation, and IL-1β release in bacterial infected macrophages (Gomes et al., 2021). In addition, this inflammatory profile induced by STING is associated with the control of *Brucella* persistence since HIF-1α-deficient animals are more susceptible to bacterial infection (Gomes et al., 2021). HIF-1α stabilization induced by STING during *B. abortus* infection is influenced by mitochondrial reactive oxygen species (mROS) production. Additionally, STING elicits the production of the metabolite succinate in infected macrophages. Succinate leads to HIF-1α stabilization and IL-1β secretion as shown in Figure 1C. Our findings demonstrate the mechanisms by which STING induces metabolic reprogramming in infected macrophages *via* the HIF-1α pathway.

The mitochondrial enzyme aconitate decarboxylase 1 (ACOD1, also termed as IRG1) is involved in itaconate production and function as potential modulator of cell metabolism. Bacterial LPS induces *ACOD1* gene expression in macrophages (Lee et al., 1995). In turn, ACOD1-activaded macrophages produces itaconate with potential anti-inflammatory activity (Bambouskova et al., 2021). Chen et al. (2022) studies using the sepsis model reported that STING mediates LPS-induced ACOD1 expression by binding to MyD88. They showed that the STING-MyD88 pathway mediates inducible ACOD1 expression in macrophages activated by TLR1, TRL2, TRL4, TRL5, or TLR6 ligands. Overall, activated STING in monocytes and macrophages interacts with MyD88 leading to LPS-induced ACOD1 expression and itaconate production that will result in septic death in host cells. So, far this connection between STING and MyD88 signaling pathway during *Brucella* infection is not yet understood. However, it is possible that STING-MyD88 hub is important to drive host cells to a metabolic state sufficient to trigger inflammatory responses and bacterial infection control.

## Final considerations

Recently, studies have connected cell metabolism to innate immune activation. Metabolic shift in immune cells can occur to drive inflammatory or anti-inflammatory profiles. Inflammatory signals will lead to a metabolic switch in innate immune cells resulting in aerobic glycolysis. A landmark of pharmaceutical intervention has arisen with a concept of reprogramming immune cells by changing the metabolic profile using small molecules and metabolites. In this opinion article, we tried to connect innate immune sensors responsible for *Brucella* recognition and host protection and how the activation of these receptors and adaptor molecules result in metabolic shift in macrophages. More data are reported for TLR-MyD88 and STING pathways; however, scarce information is available for the AIM2 inflammasome association with metabolic reprogramming of host cells. The findings reported here highlight the potential use of metabolites such as succinate and itaconate to combat bacterial infections like Brucellosis. More pre-clinical and clinical investigation are required to determine the role of metabolites during the crosstalk between innate immune cells and metabolic pathways. In summary, we discussed here the recent developments in the metabolic reprogramming of macrophages and speculate on the prospect of targeting immunometabolism in the effort to develop novel therapeutics to treat *Brucella* and other bacterial infections.

## Author contributions

SO and EG drafted, critically revised the manuscript, and agree to be accountable for the content of the work. All authors contributed to the article and approved the submitted version.

## Funding

This work was funded by Conselho Nacional de Desenvolvimento Científico e Tecnológico (grant#303044/ 2020-9), Fundacão de Amparo a Pesquisa do Estado de Minas Gerais (grants# APQ #01945/17 and Rede #00140-16), and National Institutes of Health (grant# R01 AI116453).

## Conflict of interest

The authors declare that the research was conducted in the absence of any commercial or financial relationships that could be construed as a potential conflict of interest.

## Publisher's note

All claims expressed in this article are solely those of the authors and do not necessarily represent those of their affiliated organizations, or those of the publisher, the editors and the reviewers. Any product that may be evaluated in this article, or claim that may be made by its manufacturer, is not guaranteed or endorsed by the publisher.
